# Nestin prevents mesenchymal stromal cells from apoptosis in LPS-induced lung injury via inhibition of unfolded protein response sensor IRE1α

**DOI:** 10.1093/lifemedi/lnac049

**Published:** 2022-11-04

**Authors:** Hongmiao Wang, Chenhao Jiang, Jianye Cai, Qiying Lu, Yuan Qiu, Yi Wang, Yinong Huang, Yong Xiao, Boyan Wang, Xiaoyue Wei, Jiahao Shi, Xingqiang Lai, Tao Wang, Jiancheng Wang, Andy Peng Xiang

**Affiliations:** Centre for Stem Cell Biology and Tissue Engineering, Key Laboratory for Stem Cells and Tissue Engineering, Ministry of Education, Sun Yat-sen University, Guangzhou 510080, China; Centre for Stem Cell Biology and Tissue Engineering, Key Laboratory for Stem Cells and Tissue Engineering, Ministry of Education, Sun Yat-sen University, Guangzhou 510080, China; Department of Hepatic Surgery and Liver Transplantation Centre, The Third Affiliated Hospital, Sun Yat-sen University, Guangzhou 510630, China; Guangdong Key Laboratory of Liver Disease Research, Guangdong Engineering Laboratory for Transplantation, The Third Affiliated Hospital, Sun Yat-sen University, Guangzhou 510630, China; Centre for Stem Cell Biology and Tissue Engineering, Key Laboratory for Stem Cells and Tissue Engineering, Ministry of Education, Sun Yat-sen University, Guangzhou 510080, China; Centre for Stem Cell Biology and Tissue Engineering, Key Laboratory for Stem Cells and Tissue Engineering, Ministry of Education, Sun Yat-sen University, Guangzhou 510080, China; Guangdong Institute for Drug Control, NMPA Key Laboratory for Quality Control of Blood Products, Guangdong Drug Administration Key Laboratory of Quality Control and Research of Blood Products, Guangzhou 510663, China; Department of Endocrinology, The First Affiliated Hospital of Sun Yat-Sen University, Guangzhou 510080, China; Centre for Stem Cell Biology and Tissue Engineering, Key Laboratory for Stem Cells and Tissue Engineering, Ministry of Education, Sun Yat-sen University, Guangzhou 510080, China; Centre for Stem Cell Biology and Tissue Engineering, Key Laboratory for Stem Cells and Tissue Engineering, Ministry of Education, Sun Yat-sen University, Guangzhou 510080, China; Centre for Stem Cell Biology and Tissue Engineering, Key Laboratory for Stem Cells and Tissue Engineering, Ministry of Education, Sun Yat-sen University, Guangzhou 510080, China; Centre for Stem Cell Biology and Tissue Engineering, Key Laboratory for Stem Cells and Tissue Engineering, Ministry of Education, Sun Yat-sen University, Guangzhou 510080, China; Centre for Stem Cell Biology and Tissue Engineering, Key Laboratory for Stem Cells and Tissue Engineering, Ministry of Education, Sun Yat-sen University, Guangzhou 510080, China; Centre for Stem Cell Biology and Tissue Engineering, Key Laboratory for Stem Cells and Tissue Engineering, Ministry of Education, Sun Yat-sen University, Guangzhou 510080, China; Centre for Stem Cell Biology and Tissue Engineering, Key Laboratory for Stem Cells and Tissue Engineering, Ministry of Education, Sun Yat-sen University, Guangzhou 510080, China; Scientific Research Centre, The Seventh Affiliated Hospital, Sun Yat-sen University, Shenzhen 518107, China; Centre for Stem Cell Biology and Tissue Engineering, Key Laboratory for Stem Cells and Tissue Engineering, Ministry of Education, Sun Yat-sen University, Guangzhou 510080, China

**Keywords:** acute respiratory distress syndrome, LPS-induced lung injury, ER stress, IRE1α, Nestin

## Abstract

The clinical applications of MSC therapy have been intensely investigated in acute respiratory distress syndrome. However, clinical studies have fallen short of expectations despite encouraging preclinical results. One of the key problems is that transplanted stem cells can hardly survive in the harsh inflammatory environment. Prolonging the survival of transplanted MSCs might be a promising strategy to enhance the therapeutic efficacy of MSC therapy. Here, we identified Nestin, a class VI intermediate filament, as a positive regulator of MSC survival in the inflammatory microenvironment. We showed that Nestin knockout led to a significant increase of MSC apoptosis, which hampered the therapeutic effects in an LPS-induced lung injury model. Mechanistically, Nestin knockout induced a significant elevation of endoplasmic reticulum (ER) stress level. Further investigations showed that Nestin could bind to IRE1α and inhibit ER stress-induced apoptosis under stress. Furthermore, pretreatment with IRE1α inhibitor 4μ8C improved MSC survival and improved therapeutic effect. Our data suggests that Nestin enhances stem cell survival after transplantation by inhibiting ER stress-induced apoptosis, improving protection, and repair of the lung inflammatory injury.

## Graphical Abstract

**Graphical Abstract:**
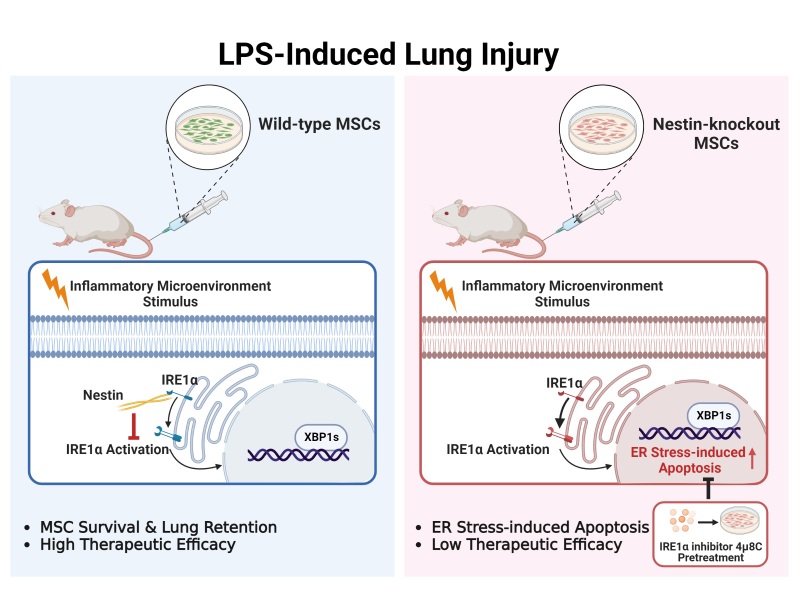

## Introduction

Acute respiratory distress syndrome (ARDS) is an acute inflammatory disease characterized by refractory hypoxia and pulmonary edema. In severe cases, acute respiratory failure and septic shock might occur, resulting in ARDS, which has a high hospital mortality rate of 46.1% [1]. Current ARDS management mainly consists of supportive care, including mechanical ventilation and extracorporeal lung care. Despite advances in supportive care within the past several decades, the therapeutic targets involved in the pathogenesis, resolution, and repair of ARDS remain unclear [2]. There is no approved causative pharmacotherapy for ARDS. As a consequence, development of efficacious therapeutic strategies for ARDS is urgently needed.

Mesenchymal stem cells (MSCs) are nonhemopoietic stromal cells that were originally isolated from bone marrow. Subsequently, these cells were found to be widely distributed in various mesenchymal tissues, such as adipose tissue, placenta, and umbilical cord [3]. Several properties make MSCs an attractive candidate for cell therapy in treating acute diseases such as ARDS [4]. MSCs exert immunomodulatory and anti-inflammatory effects both *in vitro* and *in vivo.* Once separated from host tissue, these cells can be expanded rapidly *ex vivo.* MSCs are regarded as nonimmunogenic due to the low expression level of MHC type I [5]. Based on these properties, the therapeutic potential of MSCs in ARDS has been explored in several preclinical studies in animals and *ex vivo* human lung preparations [6, 7]. Since 2014, several clinical trials have demonstrated the safety profile of MSC therapy in patients with ARDS and sepsis [8, 9]. In a randomized phase IIa START study (NCT02097641), MSC therapy was proven to be well tolerated in patients with moderate to severe ARDS. However, clinical outcomes showed no significant improvement in the MSC group, although post hoc analyses showed a trend for improvement in the oxygenation index. Furthermore, as the post-thaw viability of MSCs ranged from 36% to 85%, the authors proposed that the survival of transplanted MSCs could determine the clinical outcome of MSC therapy [10]. Therefore, increasing the survival rate of transplanted MSCs in the inflammatory microenvironment might be a new approach to improve therapeutic effects.

The endoplasmic reticulum (ER), the organelle that mainly facilitates protein synthesis, is tightly related to protein quality control and cell survival [11, 12]. Under stress conditions, unfolded and misfolded peptides accumulate in the ER, termed ER stress, which triggers the unfolded protein response (UPR) to resolve protein misfolding [13, 14]. Nevertheless, when the UPR is insufficient to alleviate ER stress, prolonged activation of the UPR activates the apoptotic cascade in an attempt to preserve whole-tissue integrity [15]. Hyperactivation of the UPR has been shown to facilitate pancreatic β cell apoptosis, which leads to type 1 diabetes [16]. However, it remains unclear whether ER stress-induced apoptosis affects MSC survival.

Nestin belongs to the intermediate filament (IFs) family that contribute to cytoskeleton formation. Nestin was originally found in the CNS stem cells [17]. Subsequently, it was found to be a marker for several stem cells, including MSCs [18], stem leydig cells [19], etc. Recently, we and other researchers have shown that Nestin could participate in the stress response in stem/progenitor cells [20]. Adrenocortical Nestin-positive cells exhibited a high migratory phenotype and can differentiate into steroidogenic cells in a stress model [21]. Similarly, we showed that Nestin knockout causes neural stem cell pool loss and subsequent embryonic lethality [22]. In addition, Nestin mediates redox homeostasis by regulating the Keap1/Nrf2 signaling pathway [23]. Eriksson et al. also found that Nestin could protect neuronal progenitor cells from exogenous oxidant-induced cell death [24]. Together, these studies indicate that Nestin could serve as an integrated intracellular stress sensor. However, whether Nestin participates in regulating ER stress in MSCs remains unknown.

In this study, we sought to discover the key mediators in maintaining MSC survival in the ARDS microenvironment. We showed that Nestin knockout significantly hampered the MSC therapeutic effect in an LPS-induced lung injury model, which could be related to the increased apoptosis level. Then, we showed that Nestin could act as a stress sensor and protect MSCs from ER stress-induced apoptosis. Mechanistically, Nestin could directly bind to the cytoplasmic region of IRE1α, one of the key components of the UPR, and inhibit its downstream signaling pathway. Furthermore, pretreatment with the IRE1α inhibitor 4μ8C prevented apoptosis of Nestin-knockout MSCs and restored the therapeutic effect.

## Results

### Nestin knockout in iPSC-derived MSCs hampers the therapeutic effect in an LPS-induced lung injury model

**Figure 1.**
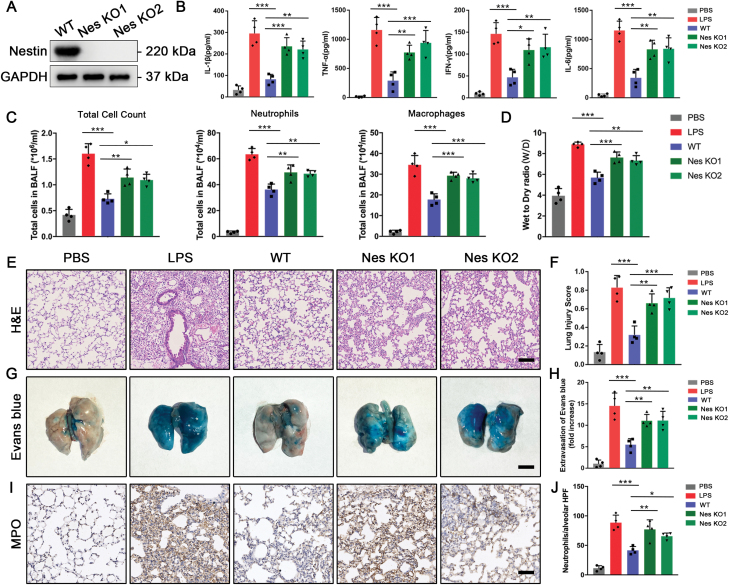
**Nestin knockout in iPSC-derived MSCs hampers the therapeutic effect in LPS-induced lung injury model.** Nestin was knocked out using CRISPR/Cas9 (Nes KO1 and Nes KO2), whole-cell extracts were prepared and the expression level of Nestin was analyzed using Western blot. (B) IL-1β, TNF-α, IFN-γ, and IL-6 in the BALF were detected by ELISA (*n* = 4). (C) Total cell count, neutrophils, and macrophages in BALF were measured. (D) Wet-to-dry ratio (W/D) was measured. (E and F) Representative images of lung sections with H&E staining. Lung injury scores were calculated. Scale bar: 100 μm. (G and H) Representative images of the lungs stained with Evans blue dye by alveolar leakage. Scale bar: 5 mm. Quantitative spectro-photometric analysis of Evans blue-labeled albumin extravasation. (I and J) Nestin-knockout MSCs administration increased alveolar neutrophil counts, revealed by myeloperoxidase (MPO) staining. Scale bar: 100 μm. The data are presented as the means ± SD. *P < 0.05, **P < 0.01, and ***P < 0.001, Student’s *t*-test.

We previously identified Nestin as a key regulator in maintaining cell homeostasis [25–27]. Therefore, to investigate whether Nestin could affect the MSC therapeutic effect, we generated two Nestin-knockout human induced pluripotent stem cell lines (hiPSCs) through CRISPR/Cas9 gene editing technology (Figs. 1A and S1A–S1D). We then induced the Nestin-knockout hiPSCs to differentiate into MSCs (Nes KO1 and Nes KO2) via a neuromesodermal progenitor intermediate based on a previously established induction system (Fig. S1E) [28]. Then, we validated that the hiPSC-derived MSCs possessed the characteristics of MSCs, including MSC surface markers and osteogenic, adipogenic, and chondrogenic trilineage differentiation properties (Fig. S1F and S1G).

To evaluate the role of Nestin in MSC therapy, we established a mouse model of lung injury by nasal instillation of lipopolysaccharide (LPS) in C57Bl/6J mice. Wild-type MSCs (WT) and Nestin-knockout MSCs were intravenously administered 4 h after injury. After 24 h, the mice were sacrificed to evaluate the lung injury level. The results showed that while the WT MSCs could ameliorate lung inflammation, the Nestin-knockout MSCs could not exert similar effects, as revealed by an elevation of the concentrations of BALF (bronchoalveolar lavage fluid) inflammatory cytokines, including IL-1β, TNF-α, IFN-γ, and IL-6 (Fig. 1B). The total cell count, neutrophil count, and macrophage count in BALF were also increased in the Nestin-knockout groups, indicating a more severe inflammation level (Fig. 1C). Furthermore, the wet-to-dry ratio significantly increased in the Nestin-knockout groups, indicating a more severe lung edema status (Fig. 1D). Consistently, we observed a worsened disruption of alveolar epithelial (Fig. 1E and 1F) and endothelial barriers (Fig. 1G and 1H) in the Nestin-knockout groups by H&E staining and Evans blue dye. In addition, neutrophil recruitment in the alveolar compartment of the Nestin-knockout groups was significantly increased (Fig. 1I and 1J). Moreover, to avoid cross-species artifact by transplanting hiPSC-derived MSCs into murine models, we silenced Nestin expression in a murine bone marrow-derived MSC (BM-MSC) cell line (shNES#1, shNES#2) (Fig. S2A). Similar results were observed in BALF (Fig. S2B and S2C), H&E staining (Fig. S2D and S2E), Evans blue staining (Fig. S2F and S2G) as well as MPO staining (Fig. S2H and S2I). These results showed that Nestin knockout hampered the therapeutic effect of MSCs within the inflammatory microenvironment.

### Nestin knockout increases MSC apoptosis in the LPS-induced lung injury model

**Figure 2.**
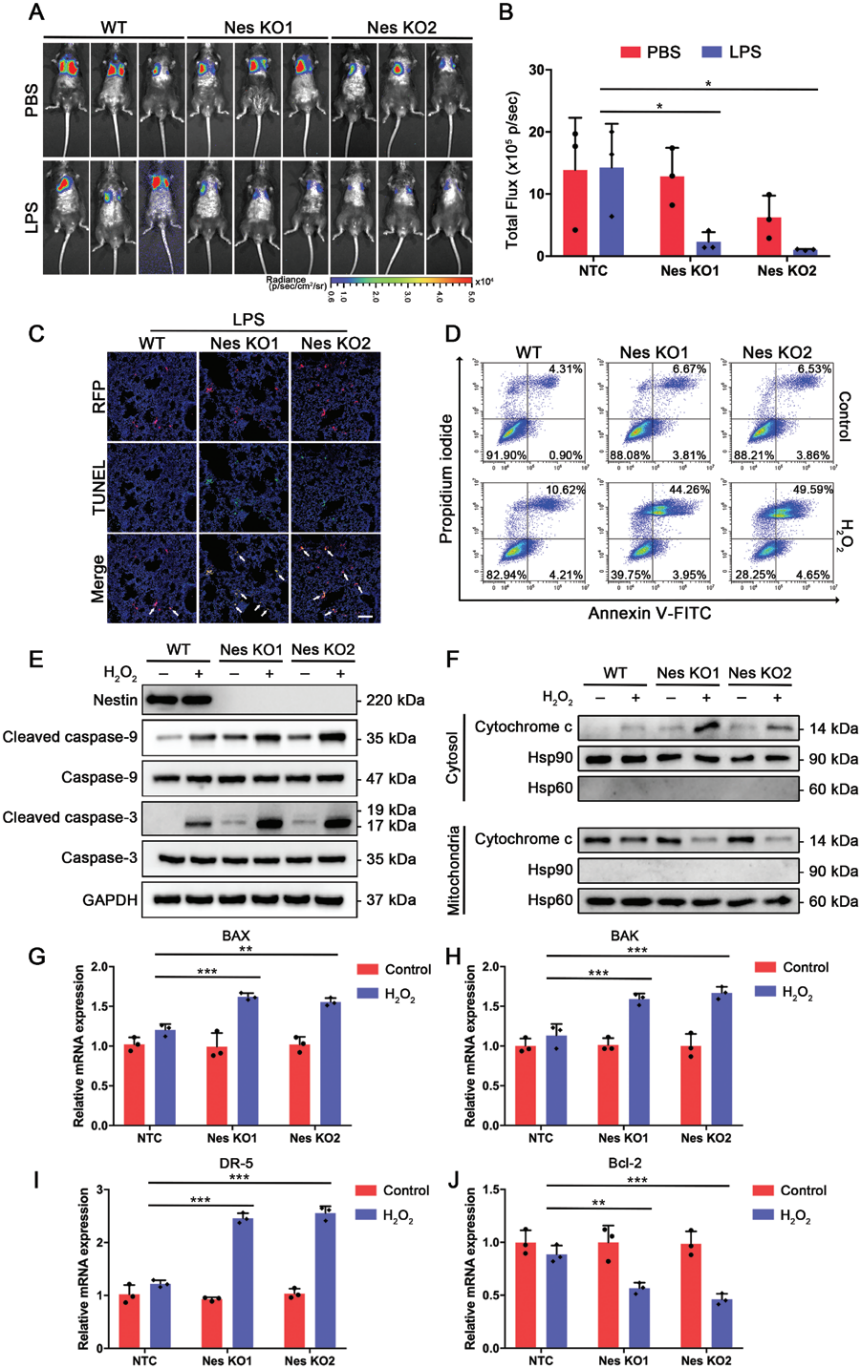
**Nestin knockout increases MSC apoptosis in LPS-induced lung injury model.** (A and B) Transplanted infused MSC^Luc^ was tracked by bioluminescence imaging after 24 h (*n* = 3). Quantifications were shown by total flux level. (C) TUNEL staining was performed on lung sections of mice transplanted with wild-type MSCs or Nestin-knockout MSCs. TUNEL-positive cells were counted from 4 random HPF per section. (D) Flow cytometric detection using Annexin V-FITC and PI to detect H_2_O_2_-induced cells death. (E) Caspase activation was evaluating using Western blot in each group under H_2_O_2_ stimulation. (F) Level of cytoplasmic Cytochrome c was detected by Western blot. (G–J) Relative mRNA fold change of pro-apoptotic genes BAX, BAK, DR5, and anti-apoptotic gene Bcl-2. The data are presented as the means ± SD. *P < 0.05, **P < 0.01, and ***P < 0.001, Student’s *t*-test.

Next, we investigated the specific mechanism through which Nestin knockout hampered the therapeutic effect. It has been suggested that the main obstacle in the clinical translation of MSC therapy is the poor survival of transplanted MSCs in the pathological microenvironment [29]. Therefore, we detected the number of MSCs within the lung tissues. First, we transfected MSCs with a luciferase vector for subsequent *in vivo* tracking. We found that Nestin knockout did not affect the number of MSCs in the lungs of healthy mice. However, in the LPS-injured mice, Nestin knockout significantly reduced the number of MSCs within the lung tissues. These results indicated that while the MSC lung entrapment level was not affected, Nestin knockout significantly hampered the survival of transplanted MSCs in the inflammatory microenvironment, which resulted in a significant reduction in MSC number (Fig. 2A and 2B). To further visualize the transplanted MSCs in the lung tissues using immunofluorescence, we further transfected MSCs with an RFP lentivirus vector (MSC^RFP^) (Fig. S3A and S3B). Subsequently, using TUNEL staining, we tested whether the Nestin-knockout MSCs underwent cell apoptosis. The results showed that Nestin knockout led to an increase in the apoptosis level of MSCs in the inflammatory microenvironment (Figs. 2C and S3C). To further explore the specific mechanism of Nestin in the progression of MSC apoptosis under stress conditions, we introduced an *in vitro* stress model generated by H_2_O_2_ addition. Similarly, we found that cell survival was markedly decreased in the Nestin-knockout groups after H_2_O_2_ treatment (Fig. S3D). In addition, H_2_O_2_ treatment caused a significant increase in cellular ROS levels (Fig. S3E and S3F). Furthermore, we used Annexin V/propidium iodide (PI) flow cytometric analysis to measure the effect of Nestin knockout on cell apoptosis. Similar to the *in vivo* results, while Nestin knockout alone had little effect on the apoptotic rate, H_2_O_2_ addition significantly increased the apoptotic rate in the Nestin-knockout groups (Figs. 2D and S3G). Besides, we showed that H_2_O_2_ treatment induced higher levels of activated caspase-9 and caspase-3 in the Nestin-knockout groups (Fig. 2E) and an increased level of cytoplasmic Cytochrome c (Fig. 2F). Subsequently, we showed that the expression levels of pro-apoptotic genes were significantly increased, while anti-apoptotic gene were decreased in Nestin-knockout groups under H_2_O_2_ condition (Fig. 2G–2J). Moreover, we knocked out Nestin expression in two clinical-used MSCs cell line, human BM-MSCs, and umbilical cord-derived MSCs (Fig. S5A and S5B). Similarly, we showed that H_2_O_2_ treatment induced higher levels of activated caspase-9 and caspase-3 in the both MSC cell lines after Nestin knockout (Fig. S5C and S5D). These data suggest that Nestin mainly facilitates MSC survival under stress conditions.

### Nestin knockout activates UPR signaling via the IRE1α pathway *in vitro*

**Figure 3.**
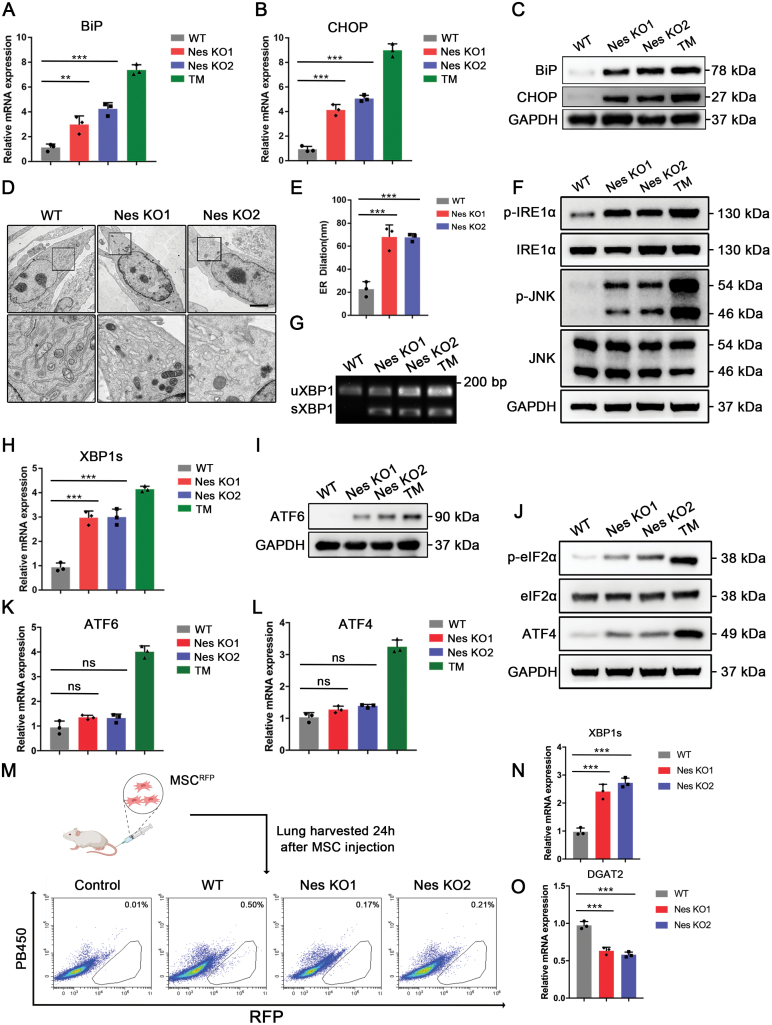
**Nestin knockout activates UPR signaling via IRE1α pathway *in vitro*.** (A and B) Relative mRNA fold change of BiP and CHOP in MSCs after Nestin knockout (*n* = 3). TM (10 μg/mL, 6 h), an ER stress inducer was used as a positive control. (C) Western blot was used to detect the levels of BiP and CHOP after Nestin knockout. (D and E) Transmission electron microscopy was used to visualize the morphology of endoplasmic reticulum in each group, images were from 3 independent repeats of cells. Scale bar: 2 μm. ER dilation were calculated for each sample. (F) Western blot was used to detect the levels of phospho-IRE1α/IRE1α and phospho-JNK/JNK. (G) The cDNA from total RNA from cells in each group were synthesized by RT-PCR. XBP1 splicing was determined by PCR using primers that amplify both spliced (XBP1s) and unspliced (XBP1u) mRNA species. (H) XBP1s mRNA was measured by qPCR. (I) Western blot was used to detect the levels of ATF6. (J) Western blot was used to detect the levels of ATF4 and its downstream phospho-eIF2α/eIF2α. (K and L) Relative mRNA fold change of ATF6 and ATF4 genes. (M) Lungs were lysed and cell suspensions were subjected to flow cytometry. (N and O) Sorted MSC^RFP^ were subjected to RT-qPCR. The data are presented as the means ± SD. *P < 0.05, **P < 0.01, and ***P < 0.001, Student’s *t*-test.

Under stress conditions, cells initiate stress response mechanisms to adapt to external cues. Among the integrated stress responses, ER stress, which regulates proper protein folding, is considered a key process [30]. Therefore, we investigated whether Nestin could regulate ER homeostasis under stress conditions. To evaluate the role of Nestin in the ER stress, we detected the expression levels of the ER stress markers immunoglobulin heavy chain-binding protein (BiP) and C/EBP-homologous protein (CHOP) following Nestin knockout. Tunicamycin (TM), a classical ER stress inducer, was used as a positive control for ER stress activation. Results showed that the expression of BiP and CHOP was significantly increased in the Nestin-knockout groups (Fig. 3A–3C). Besides, immunofluorescence staining also revealed that BiP was significantly increased and exhibited a protein aggregate structure after Nestin knockout (Fig. S4A). A similar result was observed in the transplanted MSCs within the microenvironment (Fig. S4B and S4C). As the function of the ER is closely related to its morphology, we also observed ER dilation after Nestin knockout using transmission electron microscopy (Fig. 3D and 3E). These data suggest that Nestin could regulate ER homeostasis. To further evaluate the mechanism by which Nestin regulates ER homeostasis, we measured the activation levels of the three main UPR downstream pathways (IRE1α, PERK, ATF6) to determine which mediator(s) were pivotal in UPR activation following Nestin knockout. We observed a significant elevation in phosphorylated IRE1α (p-IRE1α) and JNK (p-JNK) (Fig. 3F). Similar results were also observed in BM-MSCs and UC-MSCs (Fig. S5E–S5J). Furthermore, Nestin knockout induced XBP1 splicing, a downstream effect of IRE1α activation (Fig. 3G and 3H). In contrast, the expression levels of ATF6, ATF4, and p-eIF2α, the downstream effectors of the ATF6 and PERK signaling pathways, were only slightly altered (Fig. 3I–3L). Furthermore, we verified our findings under the LPS-induced ALI model. As expected, we found that Nestin knockout led to a decrease in the ratio of MSC^RFP^ population, compared to WT MSCs (Fig. 3M). Moreover, we demonstrated that Nestin-knockout MSCs had a higher level of IREα signaling products, such as XBP1s and a reduced level of IRE1α substrates, such as DGAT2, both suggesting the activation of IRE1α signaling pathway (Fig. 3N and 3O). Altogether, these data suggest that Nestin regulates ER stress levels mainly through the inhibition of IRE1α activation.

### Nestin directly binds to IRE1α and inhibits its subsequent signaling

**Figure 4.**
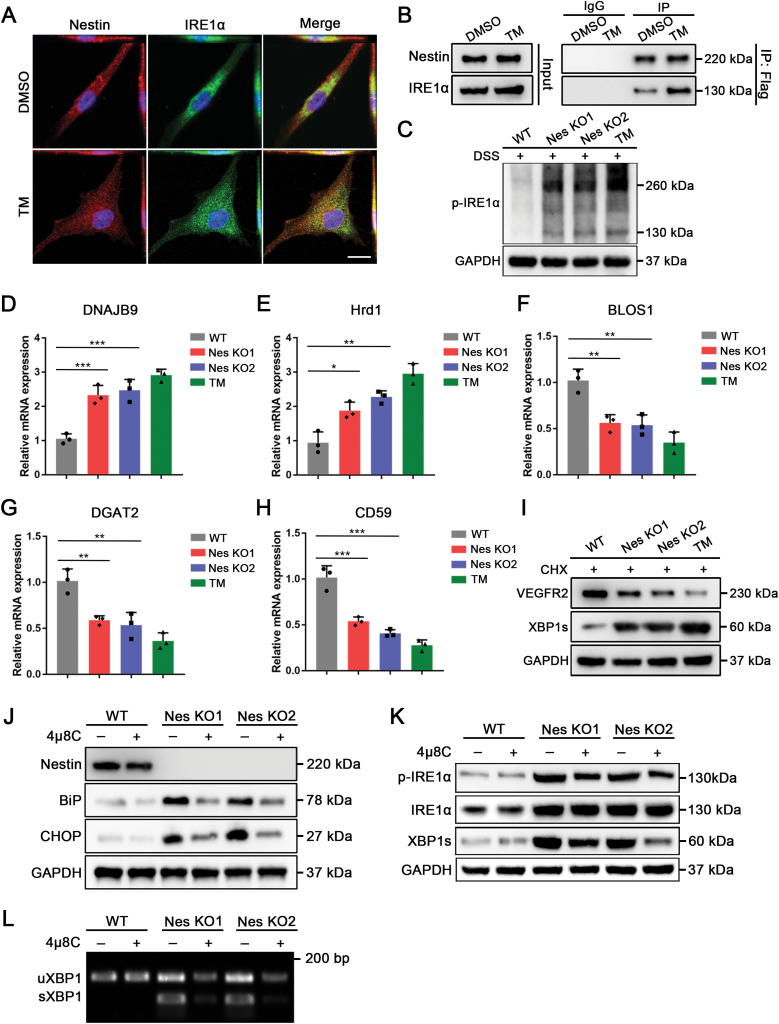
**Nestin directly binds to IRE1α and inhibits its subsequent signaling.** The co-localization of Nestin and IRE1α in MSCs under normal condition and stress condition were detected by immunofluorescence. Scale bar: 20 μm. (B) Flag-Nestin plasmid was transfected into cells, whole-cell lysates were immunoprecipitated with anti-Flag antibody, and the precipitated proteins were then blotted with the indicated antibodies. (C) Cell lysates were subjected to chemical crosslinking (XL) with disuccinimidyl suberate (DSS) to stabilize IRE1 dimers and analyzed by western blot. (D and E) Expression levels of XBP1s target genes (DNAJB9 and Hrd1) were measured by qPCR (*n* = 3). (F–H) Expression levels of RIDD target genes-BLOS1, DGAT2, and CD59 were measured by qPCR (*n* = 3). (I) Cells were treated with 50 μg/mL CHX and incubated for 1 h in each group. The levels of VEFGR2 were analyzed by western blot. (J) Western blot analysis of the levels of BiP and CHOP after 4µ8C treatment to evaluate the ER stress level. (K and L) The levels of phospho-IRE1α/IRE1α and XBP1s were determined by Western blot, XBP1 splicing was determined by PCR after 4µ8C treatment to evaluate the activation level of the IRE1 pathway. The data are presented as the means ± SD. *P < 0.05, **P < 0.01, and ***P < 0.001, Student’s *t*-test.

Next, we explored how Nestin selectively regulates IRE1α and its downstream signaling pathway. As IRE1α is a transmembrane protein localized on the ER, we hypothesized that Nestin could directly bind to the cytoplasmic domain of IRE1α. Indeed, immunofluorescence staining showed that Nestin and IRE1α colocalized throughout the cells (Fig. 4A). Co-IP assays further verified that Nestin could bind to IRE1α and that the interaction would significantly increase under stress conditions (Fig. 4B). Western blots also showed that Nestin knockout increased the level of IRE1α dimerization, which indicated an elevated IRE1α activation level (Fig. 4C). Next, we asked whether Nestin knockout could affect the downstream effects of IRE1α. As XBP1s is a well-established product of IRE1α’s RNase activity, we examined the expression of XBP1s target genes. Nestin knockout significantly increased the mRNA levels of DNAJB9 and Hrd1 (Fig. 4D and 4E). In addition, Nestin knockout significantly increased the regulated IRE1α-dependent decay (RIDD) activity of IRE1α, as revealed by decreased mRNA levels of BLOS1, DGAT2, and CD59 (Fig. 4F–4H). Furthermore, Nestin knockout increased ER-associated degradation (ERAD) activity, which led to a significant decrease in the ERAD target VEGFR2 (Fig. 4I). While Nestin knockout led to an elevation of ER stress as well as IRE1α activation, administration of the IRE1α inhibitor 4μ8C was sufficient to partially rescue this effect (Fig. 4J–4L). Further detection also revealed that 4µ8C pretreatment significantly decreased H_2_O_2_-induced cell apoptosis in the Nestin-knockout groups (Fig. S4D and S4E). Subsequently, we showed that the expression levels of pro-apoptotic genes were significantly decreased, while anti-apoptotic gene were increased in Nestin-knockout groups with 4µ8C pretreatment (Fig. S4F–S4I). Taken together, these data show that Nestin could inhibit IRE1α activity by increasing binding with the cytoplasmic region of IRE1α under stress conditions.

### Pretreatment of MSCs with the IRE1α inhibitor 4μ8C ameliorates ER stress-induced apoptosis *in vivo*

**Figure 5.**
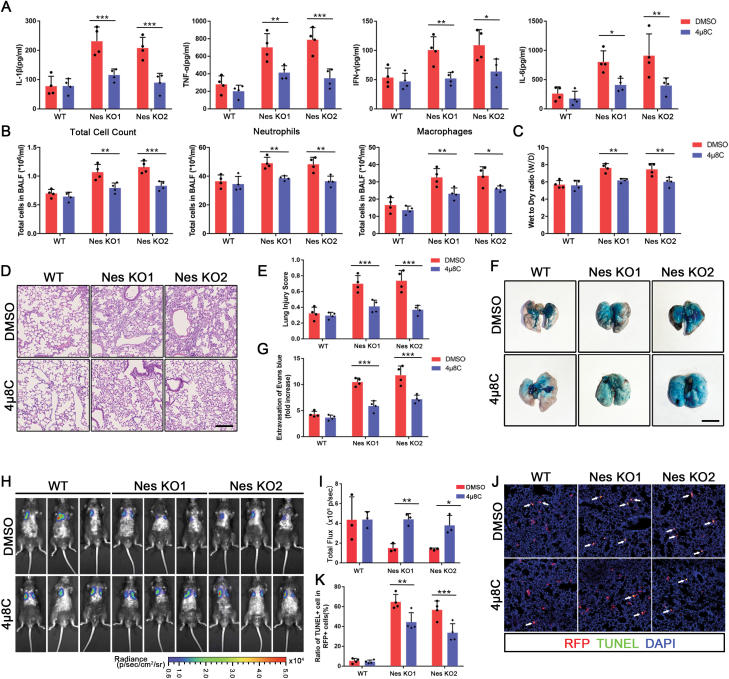
**Pretreatment of MSCs with IRE1α inhibitor 4μ8C ameliorates ER stress-induced apoptosis *in vivo*.** (A) IL-1β, TNF-α, IFN-γ, and IL-6 in the BALF were detected by ELISA (*n* = 4). (B) Total cell count, neutrophils and macrophages in BALF were measured. (C) Wet-to-dry ratio (W/D) was measured. (D and E) Representative images of lung sections with H&E staining. Lung injury scores were calculated. Scale bar: 100 μm. (F and G) Representative images of the lungs stained with Evans blue dye by alveolar leakage. Scale bar: 5 mm. Quantitative spectro-photometric analysis of Evans blue-labeled albumin extravasation. (H and I) Transplanted MSC^Luc^ was tracked by bioluminescence imaging after 24 h. Quantifications were shown by total flux level (*n* = 3). (J and K) TUNEL staining was used to observe MSC apoptotic rate in each group. TUNEL-positive cells were counted from 4 random fields per section. The data are presented as the means ± SD. *P < 0.05, **P < 0.01, and ***P < 0.001, Student’s *t*-test.

As 4µ8C pretreatment could prevent apoptosis of Nestin-knockout MSCs *in vitro*, we detected whether IRE1α inhibition could ameliorate MSC apoptosis and enhance the therapeutic effect in an LPS-induced lung injury model. Thus, we pretreated WT/Nestin-knockout MSCs with 4μ8C for 24 h before tail vein injection. The results showed that 4μ8C pretreatment reduced the expression of inflammatory factors, total cell count, neutrophil count, and macrophage count in BALF (Fig. 5A and 5B). Furthermore, 4μ8C pretreatment reduced the wet-to-dry ratio of injured lungs in the Nestin-knockout groups (Fig. 5C). In addition, the injury of the alveolar epithelial and endothelial barriers (Fig. 5D–5G), as well as neutrophil recruitment levels (Fig. S6A and S6B), was also improved in the Nestin-knockout groups after pretreatment with 4μ8C. These data showed that 4μ8C pretreatment could recover the therapeutic potential of Nestin-knockout MSCs.

Subsequently, we evaluated the number of MSCs after pretreatment with 4μ8C. As expected, we found that 4μ8C pretreatment significantly increased the number of MSCs within the lung tissues of the Nestin-knockout groups (Figs. 5H and 5I, S6C and S6D). TUNEL staining showed that 4μ8C pretreatment significantly reduced the apoptosis rate of the Nestin-knockout MSCs (Fig. 5J and 5K). Taken together, these results showed that the apoptosis of the Nestin-knockout MSCs was attenuated by pretreatment with the IRE1α inhibitor 4μ8C, which could subsequently recover the therapeutic effect of MSC therapy.

## Discussion

**Figure 6.**
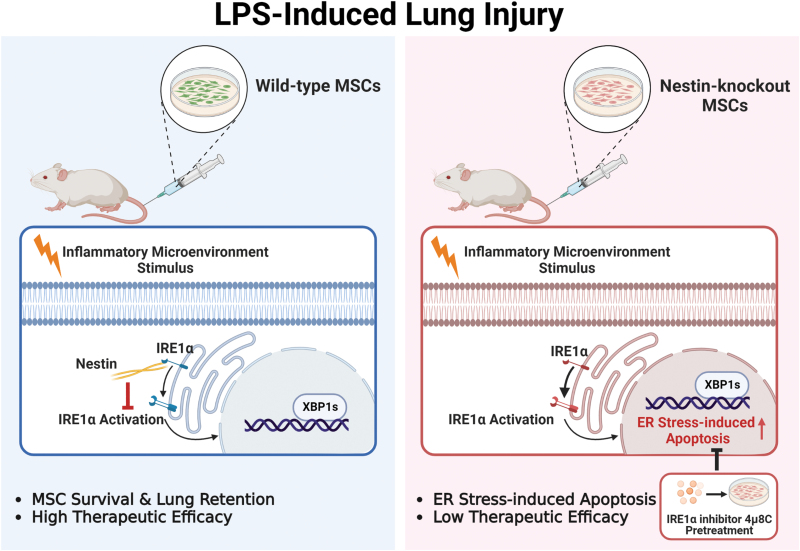
Graphical summary.

In this study, we demonstrated that (i) Nestin could maintain MSC survival within the lung inflammatory microenvironment; (ii) Nestin could bind to IRE1α and inhibit ER stress-induced apoptosis under stress; and (iii) pretreatment with the IRE1α inhibitor 4μ8C increased MSC survival and improved therapeutic effects. These findings highlight the roles of Nestin in facilitating the stress-resistance capability of MSCs and thereby enhancing the therapeutic effect of MSCs (Fig. 6).

The role of Nestin in the fate and function of stem cells has been well established. For example, during late puberty, MSCs in long bones undergo programmed senescence, characterized by loss of Nestin expression [31]. Furthermore, Nestin expression was shown to be tightly related to the differentiation stage in myogenic precursor cells [32]. Thus, we speculated that Nestin expression is related to MSC therapeutic potential. Indeed, we showed that Nestin knockout in MSCs resulted in poor survival and reduced therapeutic effects. Mechanistically, we have shown that Nestin, an IF that widely distributes in cells, could serve as an integrated homeostasis regulator through regulating mitochondria, nucleoskeleton stability, receptor trafficking [25–27]. In addition, Nestin can maintain redox homeostasis [23]. Consistently, in this study, we found that Nestin could maintain ER homeostasis by regulating UPR levels, especially through inhibition of the IRE1α signaling pathway, and subsequently maintain MSC survival in the lung inflammatory microenvironment. However, as Nestin can affect multiple organelles, early-stage apoptosis after Nestin knockout may be associated with abnormalities in multiple organelles. In addition, further investigations of whether Nestin as an integrated protector of homeostasis, could indicate an MSC subpopulation with higher therapeutic potential might be promising in the future.

Traditional theories contribute the therapeutic potential of MSCs to their paracrine effects and direct contact [4, 33, 34], which required viable MSCs to exert therapeutic effects. However, several studies demonstrated that host response to dying MSCs could also exert therapeutic effects [35]. For example, Pang et al. demonstrated that apoptotic MSCs modulated immune response through efferocytosis by macrophages in an allergic asthma model [36]. Similarly, Ko et al. showed that although engraft only transiently, MSCs confer long-term therapeutic benefits in experimental autoimmune uveitis through induction of innate immune tolerance [37]. To resolve this controversy, it has been suggested that the pathological microenvironment could determine the fate and modes of actions of transplanted MSCs [38]. For example, a preclinical study showed that MSCs were retained for longer periods in the presence of pulmonary infection than in healthy controls [39]. Moreover, recently, Rolandsson Enes et al. demonstrated that exposure of MSCs to bronchoalveolar lavage (BAL) fluid samples of ARDS patients decreased HLA-1/HLA-2 gene expressions, which facilitates host response evasion of MSCs and might extend the time of MSCs *in vivo* to exert any therapeutic effect [40]. These studies supported the concept that viable MSCs should exert therapeutic effects in ARDS/ALI microenvironment. However, the key regulators, as well as specific mechanisms through which the pathological microenvironment determines the fate and modes of action of transplanted MSCs, remains to be investigated.

In fact, poor survival of transplanted MSCs has been regarded as a major obstacle hampering the clinical translation of MSC therapy [29]. Previous studies have shown that <5% of transplanted MSCs were detectable in the injured lungs 7 days post-transplantation [41]. Therefore, to prolong MSC survival, several attempts have been made [29, 42], such as (i) genetic modifications of MSCs [43, 44]. MnSOD overexpression was reported to promote MSC engraftment and differentiation, thus attenuating acute lung injury. (ii) For preconditioning strategies, hypoxia was reported to promote MSC proliferation [45, 46], and hypoxia-preconditioned MSCs demonstrated extended survival and ameliorated lung injury [47]. In our study, we showed that pretreatment of Nestin-knockout MSCs with the ER stress inhibitor 4μ8C could inhibit ER stress-induced apoptosis in Nestin-knockout MSCs and restore the therapeutic effect. However, 4μ8C pretreatment of WT MSCs did not result in significant improvement in either MSC survival or therapeutic effects. Whether 4μ8C treatment could exhibit a similar effect in maintaining cell survival in cells with low Nestin expression levels (e.g., aged MSCs after long-term expansion) requires further investigation.

## Research limitations

In this study, we demonstrated that Nestin could maintain MSC survival within the lung inflammatory microenvironment. However, it remains to be investigated whether Nestin overexpression could further prolong the survival of transplanted MSCs and subsequently enhance the efficacy of MSC therapy. Our findings suggest that Nestin could serve as a functional marker for MSCs with higher therapeutic potential, and further clinical investigations are required to support our hypothesis.

## Materials and methods

### Animal studies

For *in vivo* experiments, 6-week-old male C57BL/6J mice were purchased from Gempharmatech, and housed under standard specific-pathogen-free conditions. For LPS-induced lung injury model, mice were anaesthetized and intra-tracheally injected with 5 mg/kg of LPS (Sigma). Four hours after LPS administration, PBS or MSCs (1 × 10^6^ cells/200 µL) were administrated via tail vein injection. Mice were sacrificed 24 h after MSC treatment for subsequent experiments. All procedures were conducted under the Sun Yat-sen University Institutional Animal Care and Use committee guidelines.

### Bioluminescence imaging

MSC number within the lung tissues was examined by bioluminescence imaging 24 h after MSC injection. d-luciferin solution (Goldbio) was prepared in PBS at 15 mg/mL. Mice were intraperitoneal injected with d-luciferin (150 mg/kg) and subjected to imaging. Images were captured using In Vivo Imaging System (PerkinElmer).

### Evans blue dye

Evans blue dye (Sigma) was injected via tail vein 4 h before sacrificing animals in order to assess endothelial leakage. Lungs were harvested and immersed in formamide at 60°C After 24 h incubation, formamide including eluted blue dye was collected and centrifuged at 12,000 *g* for 20 min. The optical density of the supernatants was determined spectrophotometrically at 620 nm. The extravasated EBD concentration in lung homogenate was calculated against a standard curve.

### Cytokine levels and cell counts in BALF

BALF was acquired via repeated intra-tracheal injection and retraction of 1 mL PBS with 100 μM EDTA for three times. Sample was centrifuged for 7 min at 400 *g* at 4°C. Then the supernatant was collected. Inflammatory cytokine levels were determined by ELISA kit (Neobioscience) according to manufacturer’s instructions. The cell pellet was harvested and counted under high power field.

### Cell culture experiments

Cells were cultured on Matrigel (BD Bioscience)-coated plates in ACF medium (Stemcell Technologies). The cells were passaged every 3 days using StemPro Accutase Cell Dissociation Reagent (Life Technologies). We utilized one hiPSC line that was established previously [48], the neuromesodermal differentiation and MSC differentiation procedure were as previously described [28]. For drug pretreatment, 4μ8C (5 μM), was added into the culture medium 24 h before harvesting MSCs for subsequent experiments.

### RNAi transfection

ShRNA transfections were performed using the MegaTran 1.0 Transfection Reagent (OriGene) according to the manufacturer’s instructions. The lentiviruses were used to infect murine-derived MSCs with Polybrene (8 μg/mL) for 4 h. The original medium was replaced with fresh medium 12 h later. The siNestin, shNES#1 (5ʹ-GGAAGAAGTTCCCAGGCTTCT-3ʹ), shNES#2 (5ʹ-GCTGAAGCTGCATTTCCTTGG-3ʹ), and their encoding vectors were transfected into MSCs using the Lipofectamine RNAiMAX Transfection Reagent (Invitrogen).

### IF

For cell immunofluorescence staining, cells were fixed with 3.7% formaldehyde for 10 min, and then permeabilized in 0.2% Triton X-100 reagent for 15 min. Samples were incubated with primary antibodies at 4°C overnight, and then treated with secondary antibodies at room temperature for 1 h in the dark. Nuclei were incubated with DAPI for 5 min. Images were acquired using LSM800 confocal microscope (Zeiss). Antibodies are listed in Table S1.

### Western blot

For immunoblotting, cells were washed with PBS for 3 times and lysed using RIPA buffer (Millipore), supplemented with protease inhibitor cocktail (Roche) and phosphatase inhibitor cocktail (Roche) for 30 min on ice. Then, cell lysates were centrifuged at 10,000 rpm for 10 min at 4°C, and blotted by SDS-PAGE at corresponding concentration and transferred to the PVDF membrane (Millipore). Targeted proteins were immunoblotted with indicated antibodies. For IRE1α dimer detection, disuccinimidyl suberate DSS (Thermo) was added to a final concentration of 2.5 mM, and incubated on ice for 2 h before protein lysis. Antibodies are listed in Table S1.

### Co-immunoprecipitation

For immunoprecipitation assay, cells were washed with PBS for 3 times and lysed using Pierce IP lysis buffer (Thermo) supplemented with protease inhibitor cocktail (Roche) and phosphatase inhibitor cocktail (Roche) for 30 min on ice. Lysates were purified by centrifugation at 10,000 rpm for 10 min at 4°C. Supernatants were incubated with indicated antibodies at 4°C overnight, followed by incubation with Protein G magnetic beads (Thermo) at 4°C for 2 h. Immunocomplexes were washed twice with IP lysis buffer for subsequent immunoblotting. Antibodies are listed in Table S1.

### RNA extraction, cDNA synthesis, and real-time quantitative PCR

Total RNA was prepared using the TRIzol reagent according to the manufacturer’s instructions. 1 μg sample was subjected to reverse transcription using a kit (Novoprotein). The generated cDNAs were used for real-time quantitative-PCR (qPCR), qPCR was performed using a 480 SYBR Green I Master Kit (Roche) and a LightCycler480 Detection System (Roche). The primer sequences used for real-time PCR are listed in Table S2.

### Lung digestion

Lungs were dissected into 1 mm^3^ pieces and digested for 1 h by Collagenase type I (Worthington) and 50 U/mL DNAse I (Sigma-Aldrich) at 37°C. The digested lung samples were passed through a 70-micron cell strainer and centrifuged. The cell pellet was resuspended in red blood cell lysis buffer, washed, and resuspended for subsequent flow cytometry analysis.

### Apoptosis assay and flow cytometry

Cells were incubated with 400 μM H_2_O_2_ for 6 h. Then, both suspended and attached cells were collected gently in 100 μL PBS and incubated with 5 μL FITC-conjugated Annexin V and 5 μL PI (Vazyme Biotech) for 10 min at room temperature in the dark. Samples were evaluated using CytoFLEX (Beckman Coulter) and the data were analyzed using the Cytexpert software (Beckman Coulter). All cells were gated and at least 20,000 cells were collected for each sample.

### XBP1 splicing assay

Total RNA isolation and cDNA synthesis was performed as described above. XBP1 cDNA was amplified by PCR with TaqMan polymerase (Thermo) using primers that could amplify both spliced and unspliced isoforms of XBP1. Amplifications were conducted as the following protocol: 95°C for 5 min, 34 cycles of 95°C for 1 min, 55°C for 1 min, 72°C for 1 min, and 5 min 72°C. Amplified DNA fragments were separated by electrophoresis on an 8% acrylamide gel and visualized by ethidium bromide (Sigma) staining. The primer sequences are listed in Table S2.

### Statistical analysis

All experiments were performed at least three times and data were expressed as means ± standard deviation (SD) unless otherwise specified. Comparisons between groups were performed using the Student’s *t*-test and one-way ANOVA. GraphPad Prism 7 Software was used for statistical analysis. A two-sided *P*-value < 0.05 was considered to be statistically significant. The level of significance is indicated as *P < 0.05, **P < 0.01, and ***P < 0.001.

## Supplementary Material

lnac049_suppl_Supplementary_Material

## Data Availability

All data, models, and materials generated or used during the study are available from the corresponding authors upon reasonable request.
