# Supplementary material for: Nestin prevents mesenchymal stromal cells from apoptosis in LPS-induced lung injury via inhibition of unfolded protein response sensor IRE1α
Source: Life Med. 2022 Nov 4;1(3):359–71. doi: 10.1093/lifemedi/lnac049 (PMC11749126; doi:10.1093/lifemedi/lnac049)
Supplement: lnac049_suppl_Supplementary_Material [file lnac049_suppl_Supplementary_Material.docx]

**Supplementary Information**

# Nestin prevents mesenchymal stromal cells from apoptosis in LPS-induced lung injury via inhibition of unfolded protein response sensor IRE1α

Hongmiao Wang^1#^, Chenhao Jiang^1#^, Jianye Cai^2,3#^, Qiying Lu^1#^, Yuan Qiu^1^, Yi Wang^4^, Yinong Huang^5^, Yong Xiao^1^, Boyan Wang^1^, Xiaoyue Wei^1^, Jiahao Shi^1^, Xingqiang Lai^1^, Tao Wang^1^, Jiancheng Wang^1,6,^*, Andy Peng Xiang^1,^*

^1^Centre for Stem Cell Biology and Tissue Engineering, Key Laboratory for Stem Cells and Tissue Engineering, Ministry of Education, Sun Yat-sen University, Guangzhou 510080, China

^2^Department of Hepatic Surgery and Liver Transplantation Centre, The Third Affiliated Hospital, Sun Yat-sen University, Guangzhou 510630, China

^3^Guangdong Key Laboratory of Liver Disease Research, Guangdong Engineering Laboratory for Transplantation, The Third Affiliated Hospital, Sun Yat-sen University, Guangzhou 510630, China

^4^Guangdong Institute for Drug Control, NMPA Key Laboratory for Quality Control of Blood Products, Guangdong Drug Administration Key Laboratory of Quality Control and Research of Blood Products, Guangzhou, China

^5^Department of Endocrinology, The First Affiliated Hospital of Sun Yat-Sen University, Guangzhou 510080, China

^6^Scientific Research Centre, The Seventh Affiliated Hospital, Sun Yat-sen University, Shenzhen, China

^#^The authors contributed equally to this study.

***Correspondence should be addressed to:**

Dr. Jiancheng Wang, Scientific Research Centre, The Seventh Affiliated Hospital, Sun Yat-sen University, Shenzhen 518000, China. Phone: 86-20-87335822, Fax: 86-20-87335858, E-mail: wangjch38@mail.sysu.edu.cn

Dr. Andy Peng Xiang, Centre of Stem Cell Biology and Tissue Engineering, Sun Yat-sen University, 74# Zhongshan 2nd Road, Guangzhou 510080, China. Phone: 86-20-87335822, Fax: 86-20-87335858, E-mail: xiangp@mail.sysu.edu.cn

**Supplementary Figures**

**
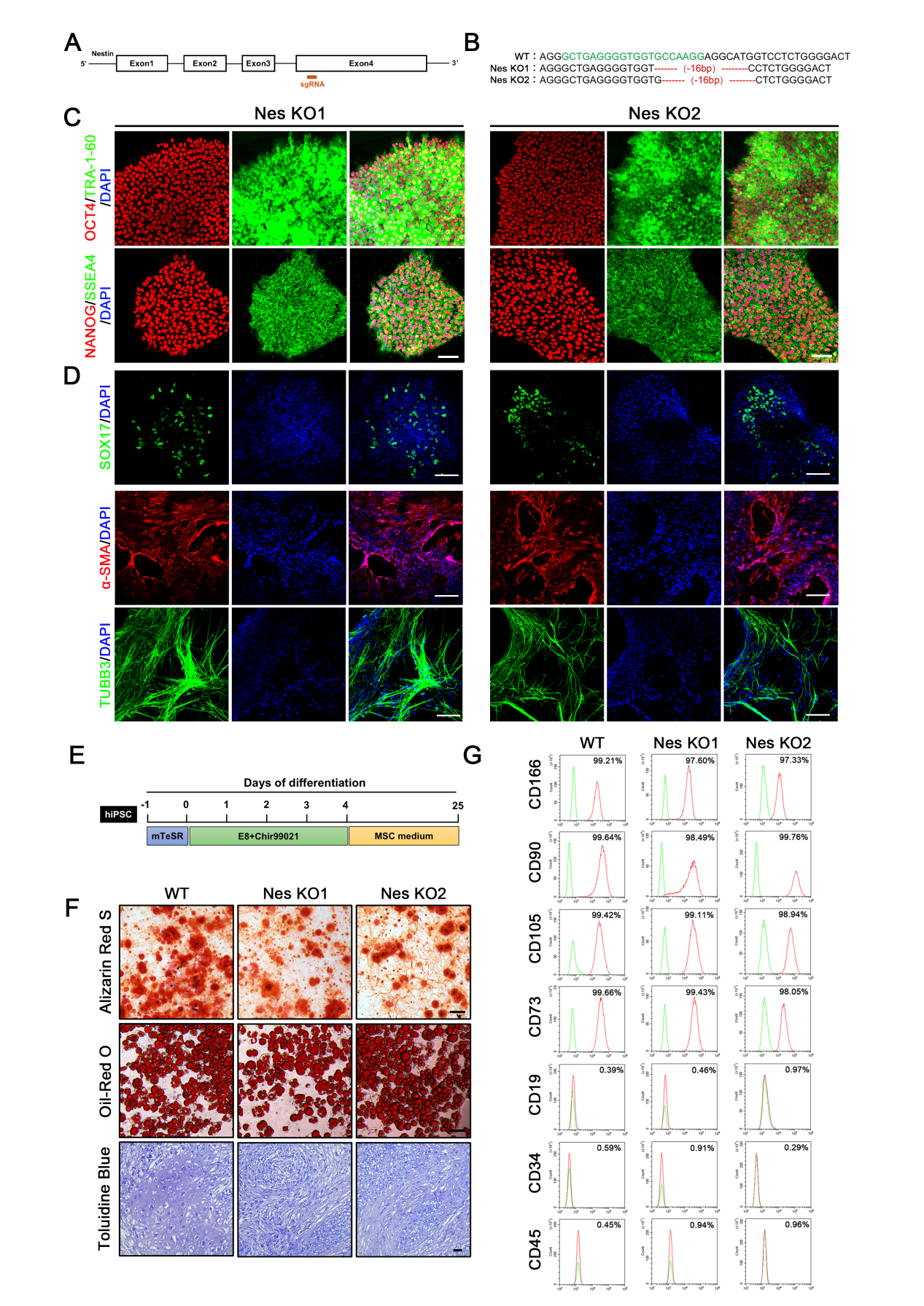
**

**Figure S1. Establishment of Nestin-knockout iPSCs with CRISPR/Cas9 technology and induction towards MSCs.**

1. Diagram showing the targets of Nestin-knockout sgRNA. (B) Genome sequencing results of Nestin-knockout cell lines. (C) Immunofluorescence staining of the pluripotency stem cell-specific markers, OCT4, TRA-1-60, NANOG and SSEA4 in Nestin-knockout hiPSCs. Scale bar: 100 μm. (D) Immunofluorescence staining of the germ layer specific markers: Endoderm (SOX17), mesoderm (α-SMA) and ectoderm (TUBB3) in Nestin-knockout hiPSCs. Scale bar: 100 μm. (E) Strategy for MSC lineage induction from hiPSC-NMP. (F) The osteogenic, adipogenic, and chondrogenic differentiation potentials of MSCs were verified by Alizarin Red S staining, oil red O staining, and toluidine blue staining, respectively. Scale bar: 100 μm. (G) FACS analysis of typical MSC surface markers in hiPSC-derived MSCs.


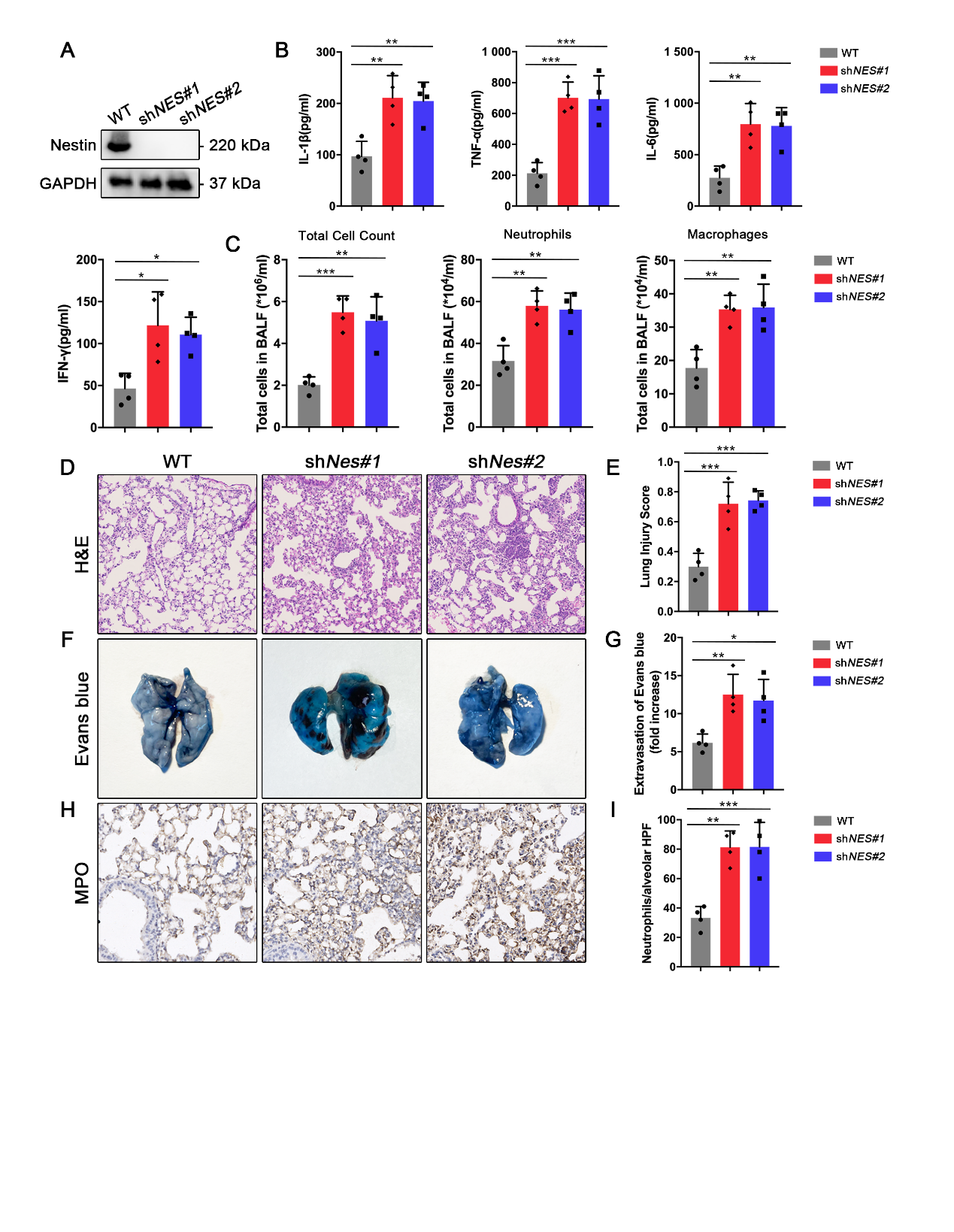


**Figure S2. Nestin deficiency in murine-derived MSCs hampers the therapeutic effect in LPS-induced lung injury model.**

(A) Nestin was silenced in murine-derived MSCs (shNES#1, shNES#2), level of Nestin was analyzed using western blot. (B) IL-1β, TNF-α, IFN-γ and IL-6 in the BALF were detected by ELISA (*n* = 4). (C) Total cell count, neutrophils and macrophages in BALF were measured. (D-E) Representative images of lung sections with H&E staining. Lung injury scores were calculated. Scale bar: 100 μm. (F-G) Representative images of the lungs stained with Evans blue dye by alveolar leakage. Scale bar: 5 mm. Quantitative spectro-photometric analysis of Evans blue-labelled albumin extravasation. (H-I) Nestin-knockout MSCs administration increased alveolar neutrophil counts, revealed by myeloperoxidase (MPO) staining. Scale bar:100 μm. The data are presented as the means ± SD. **P* < 0.05, ***P* < 0.01, and ****P* < 0.001, Student’s *t* test.


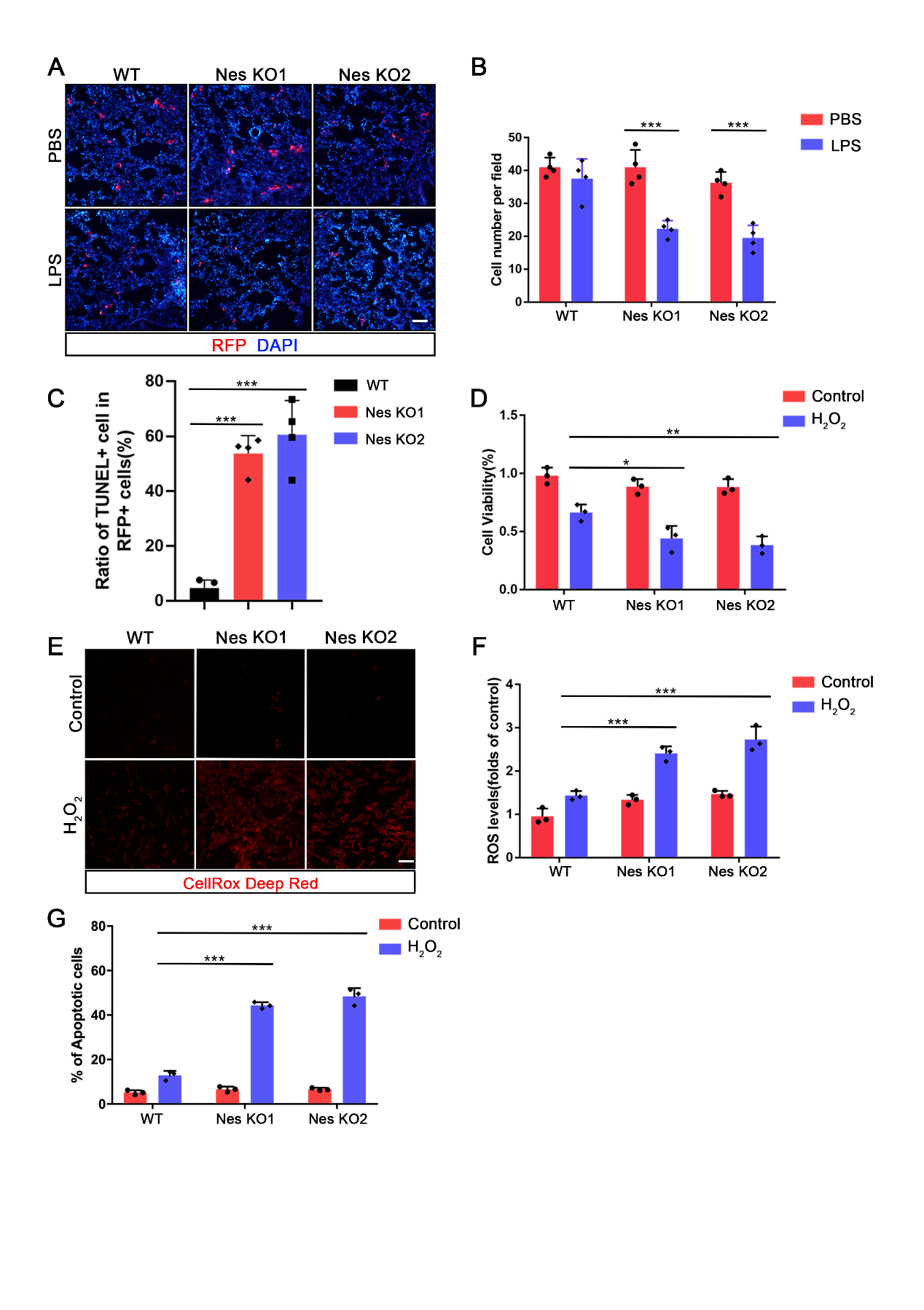


**Figure S3. Nestin knockout induced a higher apoptotic rate in MSCs under stress.**

1. B) Immunofluorescence staining was used to evaluate MSC^RFP^ colonization level in the lungs after 24 h following intravenous MSC infusion (*n* = 4). (C) TUNEL staining was performed on lung sections of mice transplanted with wild-type MSCs or Nestin-knockout MSCs. TUNEL-positive cells were counted from 4 random HPF per section. (D) Wild-type MSCs and Nestin-knockout MSCs were incubated with H_2_O_2_ (400 μM) for 6 h. Cell viability was analyzed by CCK8 assay after H_2_O_2_ treatment (*n* = 3). (E-F) Cells were treated with 400 μM H_2_O_2_ for 6 h, before using CellROX staining to evaluate ROS levels in each group (*n* = 3). Cellular fluorescence intensities were quantified. (G) Quantifications of cell apoptosis level as detected by Annexin V/propidium iodide (PI) flow cytometric analysis. The data are presented as the means ± SD. **P* < 0.05, ***P* < 0.01, and ****P* < 0.001, Student’s *t* test.


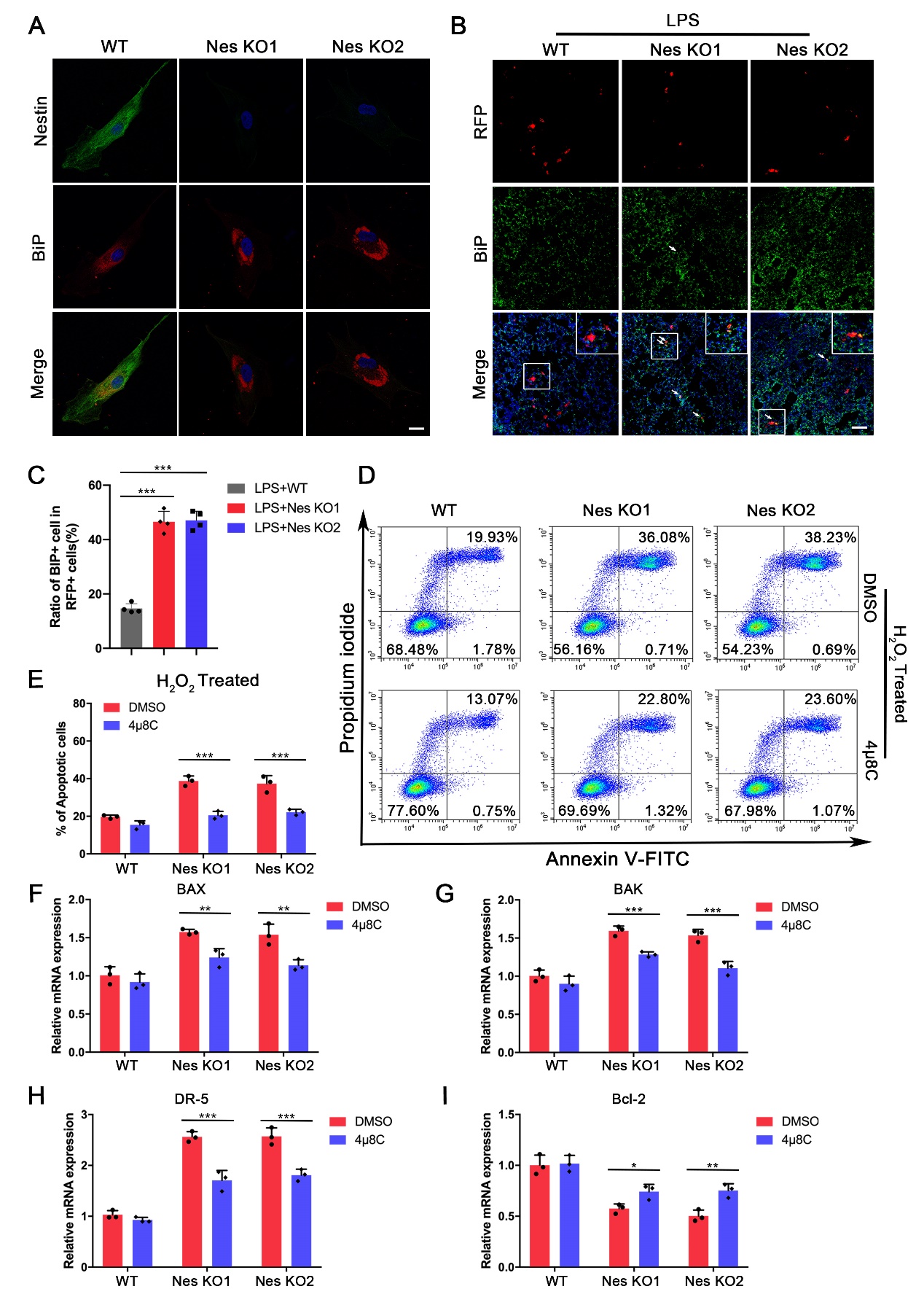


**Figure S4. Nestin knockout induced a higher UPR level under stress.**

1. Immunofluorescence staining was used to detect the morphological change of BiP aggregation in each group. (B-C) Immunofluorescence staining was used to detect the expression level of BiP in wild-type MSCs and Nestin-knockout MSCs *in vivo* (*n* = 4), Scale bar: 20 μm. (D-E) Cells were treated with 400 μM H_2_O_2_ for 6 h. Flow cytometry using Annexin V-FITC and PI was performed to detect apoptosis. Statistical analysis of the total apoptotic rate in MSCs (*n* = 3). (F-I) Relative mRNA fold change of pro-apoptotic genes BAX, BAK, DR-5, and anti-apoptotic gene Bcl-2*.* The data are presented as the means ± SD. **P* < 0.05, ***P* < 0.01, and ****P* < 0.001, Student’s *t* test.


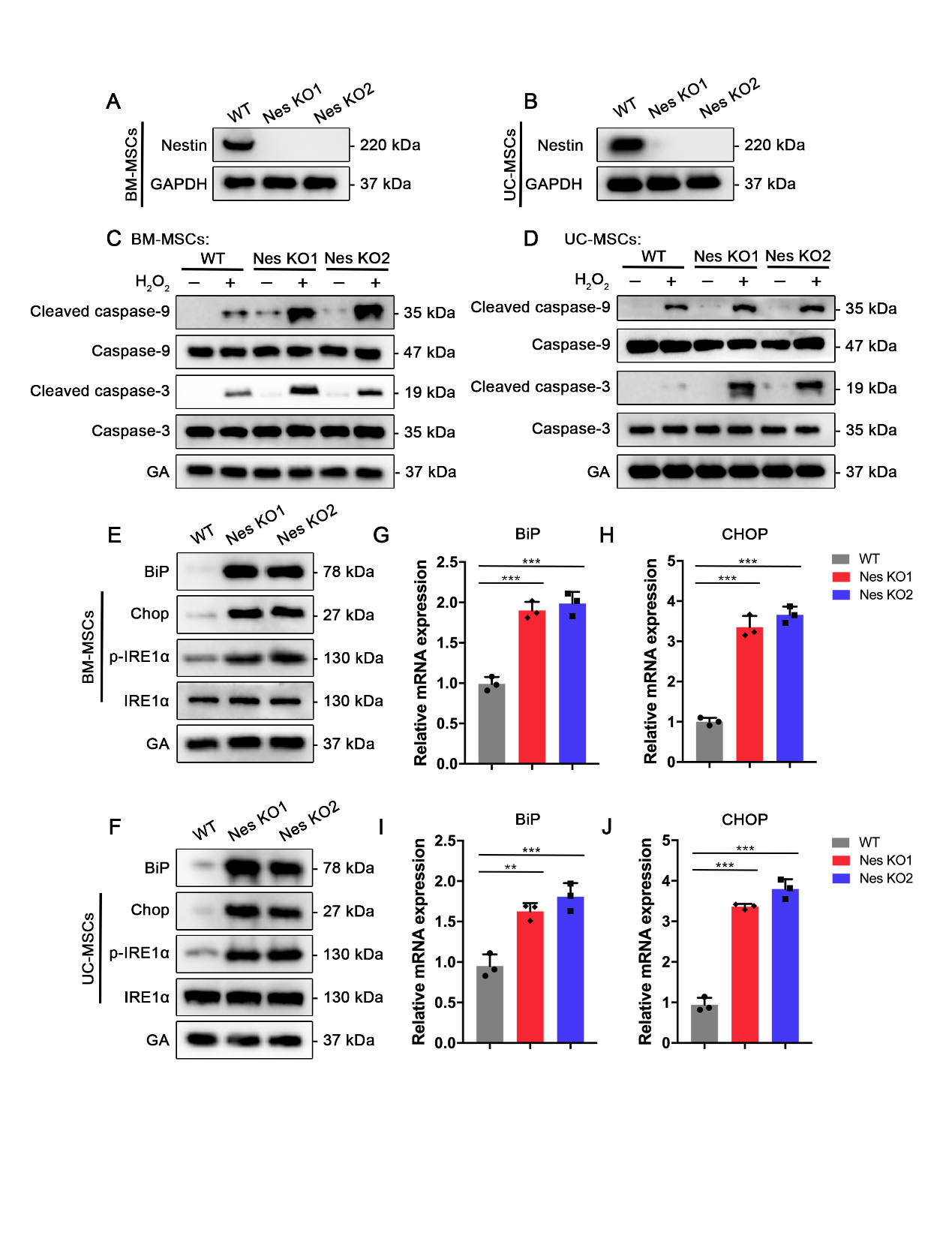


**Figure S5. Nestin knockout elevated ER stress-induced apoptosis level in BM-MSCs and UC-MSCs.**

(A-B) Nestin was knocked out using CRISPR/Cas9 (Nes KO1 and Nes KO2) in bone marrow-derived MSCs (BM-MSCs) and umbilical cord-derived MSCs (UC-MSCs), Nestin expression level was analyzed using western blot. (C-D) Caspase activation was evaluating using western blot in each group under H_2_O_2_ stimulation. (E-F) Western blot was used to detect the levels of BiP and CHOP after Nestin knockout. (G-J) Relative mRNA fold change of BiP and CHOP in BM-MSCs and UC-MSCs after Nestin knockout (*n* = 3). The data are presented as the means ± SD. **P* < 0.05, ***P* < 0.01, and ****P* < 0.001, Student’s *t* test.

**
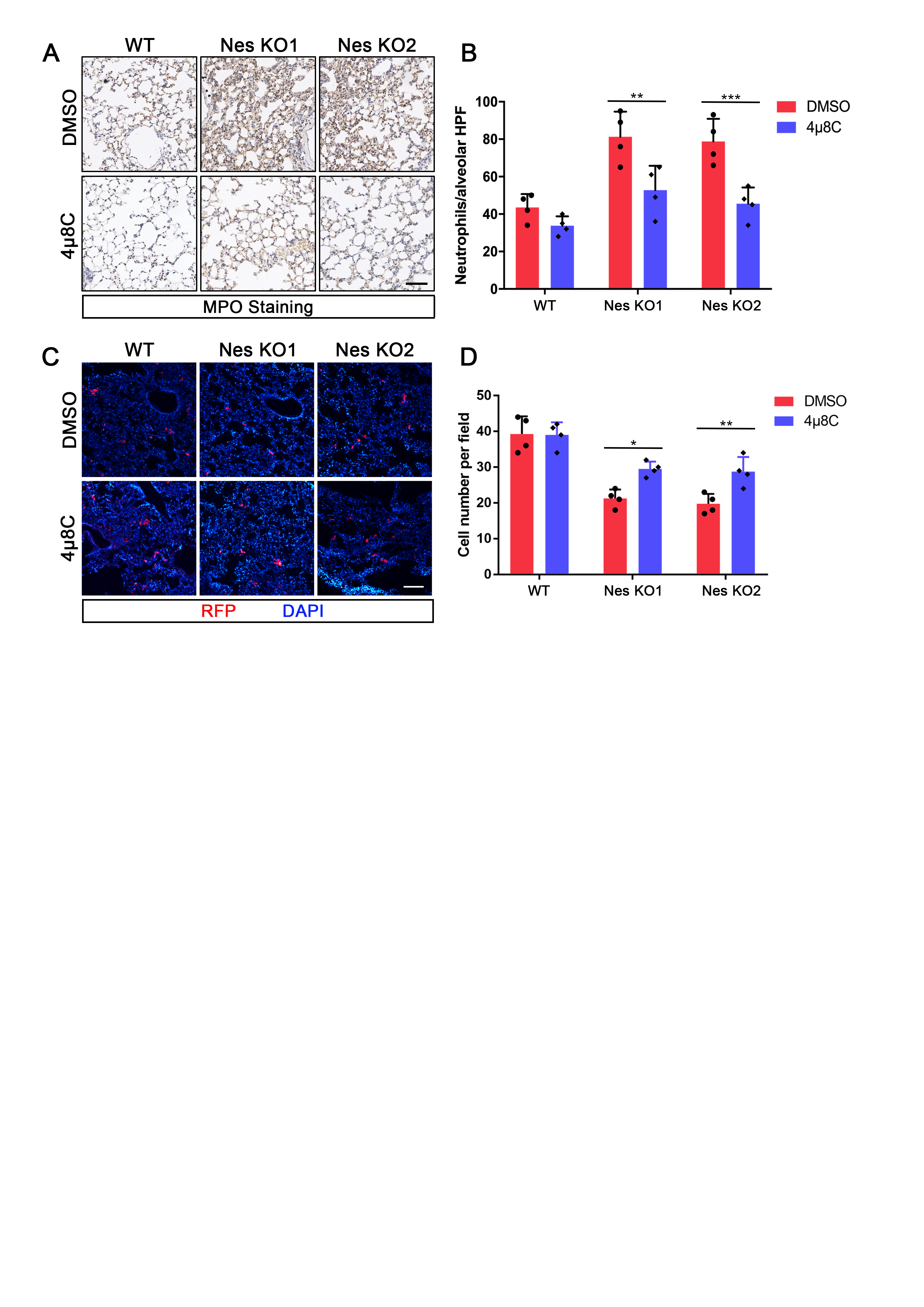
**

**Figure S6. 4μ8C pretreatment in MSCs sustained MSC survival and enhanced therapeutic efficacy.**

(A-B) MPO immuno-histochemical staining was used to detect the level of neutrophil infiltration (*n* = 4). Scale bar:100 μm. (C-D) Immunofluorescence staining was used to evaluate MSC^RFP^ colonization level in the lungs after 24 h following intravenous MSC infusion (*n* = 4). The data are presented as the means ± SD. **P* < 0.05, ***P* < 0.01, and ****P* < 0.001, Student’s *t* test.

**Table S1. Antibodies**

| **Antibody** | **Sources (Catalogue Number)** |
| --- | --- |
| **Western blot** |  |
| BiP Rabbit mAb | CST (3177) |
| CHOP Mouse mAb | CST (2895) |
| IRE1α Rabbit mAb | CST (3294) |
| Phospho-IRE1α (phospho S724) Rabbit mAb | Abcam (ab48187) |
| JNK Rabbit mAb | CST (9252) |
| Phospho-JNK (Thr183/Tyr185) Mouse mAb | CST (9255) |
| ATF6 Rabbit mAb | CST (65880) |
| ATF4 Rabbit mAb | CST (11815) |
| eIF2α Rabbit mAb | CST (9722) |
| Phospho-eIF2α (Ser51) Rabbit mAb | CST (3597) |
| VEGF Receptor 2 Rabbit mAb | CST (2479) |
| XBP1s Rabbit mAb | CST (40435) |
| Nestin mouse IgG (Western blot) | BD (611658) |
| Caspase-3 Rabbit mAb | CST (9662) |
| Cleaved caspase-3 Rabbit mAb | CST (9661) |
| Caspase-9 Rabbit mAb | CST (9504) |
| Cleaved Caspase-9 Rabbit mAb | CST (9509) |
| GAPDH Rabbit mAb | CST (2118) |
| Anti-mouse IgG HRP-linked Ab | CST (7076) |
| Anti-rabbit IgG HRP-linked Ab | CST (7074) |
| DYKDDDDK Tag Rabbit mAb | CST (14793) |
| **Immunofluorescence** |  |
| Nestin Rabbit mAb | Millipore (ABD69) |
| Nestin Mouse mAb | Millipore (MAB5326) |
| IRE1α Mouse mAb | Santa Cruz (sc-390960) |
| BiP Rabbit mAb | Abcam (ab21685) |
| goat anti-mouse IgG Alexa 488 | Invitrogen (A11001) |
| goat anti-mouse IgG Alexa 555 | Invitrogen (A21422) |
| goat anti-rabbit IgG Alexa 488 | Invitrogen (A11008) |
| goat anti-rabbit IgG Alexa 555 | Invitrogen (A21428) |
| **Immunohistochemistry** |  |
| Myeloperoxidase Rabbit mAb | Abcam (ab208670) |

**Table S2. Primers used for qPCR**

| **Name** | **Forward primer sequence** | **Reverse primer sequence** |
| --- | --- | --- |
| Nestin | 5’-CTGCTACCCTTGAGACACCTG-3’ | 5’-GGGCTCTGATCTCTGCATCTAC-3’ |
| GAPDH | 5’-CATCCTGGGCTACACTGAGC-3’ | 5’-AAAGTGGTCGTTGAGGGCAA-3’ |
| BAX | 5’-CCCGAGAGGTCTTTTTCCGAG-3’ | 5’-CCAGCCCATGATGGTTCTGAT-3’ |
| BAK | 5’-ATGGTCACCTTACCTCTGCAA-3’ | 5’-TCATAGCGTCGGTTGATGTCG-3’ |
| DR-5 | 5’-GCCCCACAACAAAAGAGGTC-3’ | 5’-AGGTCATTCCAGTGAGTGCTA-3’ |
| Bcl-2 | 5’-GGTGGGGTCATGTGTGTGG-3’ | 5’-CGGTTCAGGTACTCAGTCATCC-3’ |
| BiP | 5’-GACGGGCAAAGATGTCAGGA-3’ | 5’-GCCCGTTTGGCCTTTTCTAC-3’ |
| CHOP | 5’-AGAACCAGGAAACGGAAACAGA-3’ | 5’-TCTCCTTCATGCGCTGCTTT-3’ |
| ATF6 | 5′-GCTTTACATTCCTCCACCTCCTTG-3′ | 5′-ATTTGAGCCCTGTTCCAGAGCAC-3′ |
| ATF4 | 5’-GCTAAGGCGGGCTCCTCCGA-3’ | 5’-ACCCAACAGGGCATCCAAGTCG-3’ |
| XBP1s (qPCR) | 5’-CTGAGTCCGAATCAGGTGCAG-3’ | 5’-ATCCATGGGGAGATGTTCTGG-3’ |
| XBP1 (PCR) | 5’-TTACGAGAGAAAACTCATGGCC-3’ | 5’-GGGTCCAAGTTGTCCAGAATGC-3’ |
| DNAJB9 | 5′-GGAAGGAGGAGCGCTAGGTC-3′ | 5′-ATCCTGCACCCTCCGACTAC-3′ |
| Hrd1 | 5′-AGCTACTTCAGTGAACCCCACT-3′ | 5′-CTCCTCTACAATGCCCACTGAC-3′ |
| BLOS1 | 5′-CAAGGAGCTGCAGGAGAAGA-3′ | 5′-GCCTGGTTGAAGTTCTCCAC-3′ |
| DGAT2 | 5′-GCTGGTGCCCTACTCCAAG-3′ | 5′-CCAGCTTGGGGATGGTGA-3′ |
| CD59 | 5’-TAACCCAACTGCTGACTGCAA-3’ | 5’-TTTGGTAATGAGACACGCATCAA-3’ |

**Table S3. Reagents**

| **Reagent** | **Sources (Catalogue Number)** |
| --- | --- |
| TM (Tunicamycin) | Sigma (654380) |
| CHX (Cycloheximide) | Sigma (C7698) |
| 4μ8C | Selleck (S7272) |
| LPS (Lipopolysaccharide) | Sigma (L4524) |
| D-Luciferin | Goldbio (LUCK) |
| Annexin V-FITC/PI apoptosis kit | MultiSciences (70-AT101-100) |
| DSS (disuccinimidyl suberate) | Thermo (A39267) |
| CellROX Deep Red Reagent | Molecular Probes (C10422) |
| Mouse IL-1β ELISA Kit | Neobioscience (EMC001b) |
| Mouse TNF-α ELISA Kit | Neobioscience (EMC102a) |
| Mouse IFN-γ ELISA Kit | Neobioscience (EMC101g) |
| Mouse IL-6 ELISA Kit | Neobioscience (EMC004) |
| Evans blue dye | Sigma (E2129) |
